# Plasmon-Enhanced Ultraviolet Luminescence in Colloid Solutions and Nanostructures Based on Aluminum and ZnO Nanoparticles

**DOI:** 10.3390/nano12224051

**Published:** 2022-11-17

**Authors:** Anna A. Lizunova, Dana Malo, Dmitry V. Guzatov, Ivan S. Vlasov, Ekaterina I. Kameneva, Ivan A. Shuklov, Maxim N. Urazov, Andrei A. Ramanenka, Victor V. Ivanov

**Affiliations:** 1 Moscow Institute of Physics and Technology, National Research University, 141701 Dolgoprudny, Russia; 2 Physico-Technical Department, Yanka Kupala State University of Grodno, Ozheshko Str. 22, 230023 Grodno, Belarus; 3 B. I. Stepanov Institute of Physics, National Academy of Sciences of Belarus, Nezavisimosti Ave. 68-2, 220072 Minsk, Belarus

**Keywords:** plasmon enhanced luminescence, aluminum nanoparticles, zinc oxide nanoparticles, ultraviolet, numerical modeling, colloidal mixture, plasmon nanostructures, microplotter printing

## Abstract

Aluminum nanoparticles attract scientific interest as a promising low-cost material with strong plasmon resonance in the ultraviolet region, which can be used in various fields of photonics. In this paper, for the first time, ultraviolet luminescence of zinc oxide nanoparticles in colloid solutions and nanostructure films in the presence of plasmonic aluminum nanoparticles 60 nm in size with a metal core and an aluminum oxide shell were studied. Mixture colloids of ZnO and Al nanoparticles in isopropyl alcohol solution with concentrations from 0.022 to 0.44 g/L and 0.057 to 0.00285 g/L, correspondingly, were investigated. The enhancement of up to 300% of ZnO emission at 377 nm in colloids mixtures with metal nanoparticles due to formation of Al-ZnO complex agglomerates was achieved. Plasmon nanostructures with different configurations of layers, such as Al on the surface of ZnO, ZnO on Al, sandwich-like structure and samples prepared from a colloidal mixture of ZnO and Al nanoparticles, were fabricated by microplotter printing. We demonstrated that photoluminescence can be boosted 2.4-fold in nanostructures prepared from a colloidal mixture of ZnO and Al nanoparticles, whereas the sandwich-like structure gave only 1.1 times the amplification of luminescence. Calculated theoretical models of photoluminescence enhancement of ideal and weak emitters near aluminum nanoparticles of different sizes showed comparable results with the obtained experimental data.

## 1. Introduction

Metal nanoparticles (NPs) exhibit strong interaction with electromagnetic waves owing to a collective oscillation of the conduction band electrons, which results in remarkable absorption and scattering of light at a specific wavelength that is known as localized surface plasmon resonance (LSPR) [1,2,3,4,5,6,7,8,9,10,11,12]. This is for diverse applications such as photocatalytic pollutant degradation [2,3], solar cells [4,5], chemical and bio-sensing [6], food safety, drug discovery, and medical diagnostics [7].

The plasmon properties of nanostructures based on noble metal NPs, predominantly Ag and Au, are known to be in the visible and near infrared (NIR) spectrum region and were widely discussed in plenty of reviews [1,8,9,10]. In contrast, the interest in aluminum nanoparticles as a promising material for potential applications is still expanded due to its strong absorption in the mid–near ultraviolet (UV) range, low cost and abundance in nature [11,12]. The presence of a self-limiting natural oxide shell within the metal core ensures long-term stability [13,14] and enables a host of surface modifications such as rational ligand design for optimal dispersibility and functionality for specific applications. The surface oxide also acts as a thin dielectric film, which is ideal for nanophotonic applications for prevention of luminescence quenching [6,10,15]. The incorporation of plasmonic aluminum nanoparticles into solar cells [16,17] provides enhancement in light absorption and scattering cross-section (via LSPR) in the UV range that leads to an increase in total light conversation efficiency [18,19]. Namely, Al nanoparticles and nanolayers in UV-LED can increase their light-extraction efficiency and luminous output up to 45 % [20]. It was also shown that 3.2-fold emission enhancement at wavelengths from ∼279 to ∼286 nm can be achieved for AlGaN-based multiple quantum well structures by introducing a layer of Al NPs with sizes from 40 to 100 nm [21]. As TiO_2_ is a common semiconductor photocatalyst for organic pollutants elimination and it is photoactive only under UV light, the introduction of Al nanoparticles into the TiO_2_ films or producing specific Al/TiO_2_ nanostructures present the highest decomposition rate of malachite blue and Rhodamine B under UV light [22] compared to bare TiO_2_.

The first attempts to create surface-enhanced Raman spectroscopy (SERS) substrates based on aluminum nanoparticles showed high-potential results for applications in molecular detection. More specifically, 10^−6^ M adenine and 10^−7^ M crystal violet with an experimental enhancement factor up to 4.1 × 10^5^ were detected [23]. These findings are comparable to enhancement properties for conventional Au and Ag SERS substrates.

Zinc oxide nanoparticles are the most widespread semiconductor material for investigating the phenomena of metal-enhanced photoluminescence (PL) in the ultraviolet range near plasmonic structures based on noble metals [24] and aluminum [22,25]. Compared to raw ZnO nanorods (NRs), the UV PL intensities at 380 nm of three-dimensional ZnO NR arrays covered with layers of HfO_2_ and Al NPs were increased by approximately 16 times [26]. The enhancement factor of photoluminescence measured on an aluminum array fabricated by electron beam lithography with an Al particle size of 60 nm in diameter and 260 nm in a period with ZnO nanoparticles was only 1.5-fold [27]. On the other hand, A. Muravitskaya et al. achieved a 9.7-fold increase of the ZnO PL on the array of oval Al nanoparticles (192 × 102 nm) compared to bare ZnO nanocrystals [28]. The recent research by Qiong Ye showed 2.4 times enhancement of band-edge emission of ZnO near Al NP periodical arrays with diameter of 70 nm and period of 195 nm [29].

Development of Al-based plasmon nanostructures with required and predicted optical properties can be a challenge. Reported methods [30] of fabrication of aluminum metal-enhanced nanostructures include electron beam lithography [26,28,31] and molecular beam epitaxy [32] for production of periodically arranged nanoparticles and several techniques for the development of self-organization arrays [33] or random-packed films from colloid solutions [34,35]. The simplest method of film fabrication from colloidal nanoparticles is solvent evaporation [36], which is prone to a “coffee ring effect” associated with Marangoni flow that provides non-uniform film formation. Other versatile and accurate techniques such as dip coating [37], electrostatic deposition [38], spin coating [39], drop casting, spraying [40], inkjet [41] or aerosol printing [42,43] have been extensively employed for the preparation of monolayered or multilayered film nanostructures from liquid colloids or aerosols for plasmonic devices.

Formation of plasmonic substrates from colloidal particles as compared to lithographic approaches have several benefits such as efficient scalability, low-cost manufacturing processes and compatibility with soft materials [34]. The only required point for the colloid deposition is stable concentrated suspension of nanoparticles without agglomerates, which does not coagulate or sediment during the formation process. However, for aluminum nanoparticles passivated even with natural oxide film, it is a challenge to make specified water-based colloids due to the high reactivity and fast transformation of metal nanoparticles to aluminum hydroxide [44]. The usage of other solvents greatly limits the range of surfactants that can be used to prevent coagulation, because the main widely used stabilizing agents for aluminum oxide are insoluble in non-aqueous media. We searched the surfactants for our metal nanoparticles in the set of known agents for aluminum oxide since the surface of our metal nanoparticles was covered by an aluminum oxide shell.

It was shown that short-term stable plasmonic solutions of aluminum nanoparticles with UV absorption could be prepared by laser ablation in liquids [45,46], as well as from dry aluminum powders, synthesized by electrical explosion of the wire [14,47] and spark discharge [48], which methods have been developing in our group.

Thus, the aim of this work is to investigate plasmon-enhanced UV luminescence of ZnO nanocrystals in liquid mixtures with aluminum nanoparticles and to examine the morphological and luminescence properties of multilayer random-based ZnO and Al film nanostructures with different configurations of layers produced by microplotter printing using the obtained colloids as inks.

## 2. Materials and Methods

### 2.1. Measurement Procedure

Dynamic light scattering (DLS) measurements of hydrodynamic diameter and zeta (ζ) potential of aluminum and ZnO nanoparticles in liquid solution were carried out at 25 °C by means of Zetasizer Nano ZS (Malvern Panalytical Ltd., Malvern, UK) using disposable polystyrene cuvettes and a dip cell for ζ-potential measurements. The morphology of the NPs in the suspensions was determined by transmission electron microscopy (TEM) using a JEM-2100 microscope (JEOL, Tokyo, Japan) with an accelerating voltage of 200 kV and energy dispersive X-ray (EDX) spectrometer X-MAXN OXFORD Instruments. A few drops of the colloids were deposited on a special copper mesh with a thin amorphous carbon film in air until completely dry. In order to determine the mass fraction of NPs in the colloids, the volume of the suspension and its dry weight were measured.

The optical density spectra of the colloids in the buffer solution were measured on a JASCO V-770 (Jasco, Japan, Tsukuba) UV-Vis-NIR spectrophotometer in quartz cuvettes (Thorlabs, Newton, NJ, USA) with an optical path length of 1 cm. The measurements were made with correction on the “100% transmission” base line and with subtraction of the absorption of the solvent. The fluorescence emission spectra were measured using a JASCO FP-8300 (Jasco, Japan, Tsukuba) spectrofluorometer in quartz cuvettes (Thorlabs, Newton, NJ, USA) with an optical path length of 1 cm with excitation wavelengths of 300 and 325 nm. Mixtures of Al NPs with ZnO NPs (*v*:*v*) (1:1) were prepared at room temperature to measure FL enhancement and compared with 1:1 mixtures of ZnO NPs with pure isopropanol. The surfaces of obtained plasmon film nanostructures were studied by scanning electron microscope Jeol JSM 7001 F.

### 2.2. Preparation of Colloids and Films

**Figure 1:**
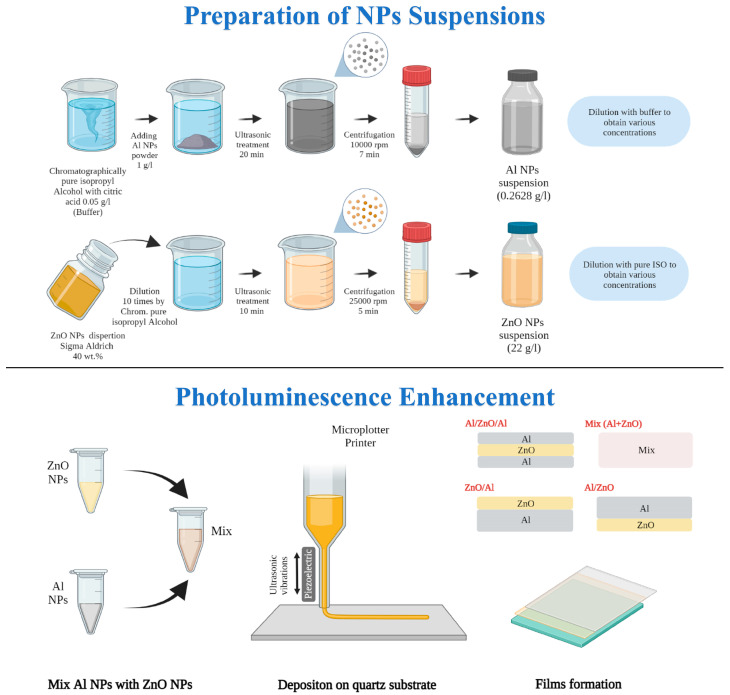
Graphical scheme of the experimental studies: colloid preparation (**top**) and fabrication of plasmon film nanostructures by microplotter printing (**bottom**).

The preparation scheme of colloids and film nanostructures is presented in Figure 1.

Al NPs Suspension: Aluminum nanopowder, produced by electrical explosion of wires [49], was used to prepare colloids of the Al NPs. A buffer solution was prepared from chromatographically pure isopropyl alcohol (Macron Fine chemicals, Avantor, Gliwice, Poland) with the addition of 0.05 g/L of citric acid (Chimmed, Moscow, Russia), which was used as stabilizer to secure aggregation and sedimentation stability for the NPs in suspensions [14]. The initial aluminum nanopowder was added to the buffer solution at a concentration of 1 g/L and submitted to ultrasonic treatment (UST) for 20 min, and subsequent accelerated sedimentation was carried out at 10,000× *g* rpm for 7 min in 20 mL metallic test tubes using a Sigma 3-30K centrifuge (Osterode am Harz, Germany). The suspension over the 14 mL of sediment from the bottom of the tube was removed. In this way, we produced as-prepared colloid of aluminum nanoparticles with a concentration 0.26 g/L. The Al NPs suspension was diluted with the required quantity of buffer. The Al NPs colloids for luminescence experiments in solutions with concentrations of 0.057, 0.0285, 0.0114, 0.0057 and 0.00285 g/L were prepared. High-concentrated aluminum colloid with a mass fraction of 0.18 g/L was used as ink for film fabrication by microplotter printing.

ZnO NPs Suspension: Zinc oxide dispersion (40 wt.%, Aldrich chemistry, Schaffhausen, Switzerland, ZnO 650 g/L) was diluted 10 times with chromatographically pure isopropyl alcohol (Macron Fine chemicals, Avantor, Gliwice, Poland) to obtain a concentration of 65 g/L. Ultrasonic treatment for 10 min and subsequent accelerated sedimentation at 25,000× *g* rpm for 5 min in 30 mL plastic test tubes were carried out. The liquid over the sediment 15 mL from the bottom of the tube was removed to produce an as-prepared ZnO colloid with a concentration of 22 g/L [50]. The ZnO NPs suspension was diluted with chromatographically pure isopropyl alcohol to obtain suspensions with concentrations of 22, 4.4, 2.2, 1.1, 0.44, 0.22, 0.044, 0.022 and 0.011 g/L.

In this work, thin-film plasmon nanostructures were deposited on the surface of a clean quartz substrate using a commercial SonoPlot GIX Microplotter II in a semi-contact printing mode. Before the experiments, quartz substrates were cleaned in piranha solution (3:1) (H_2_SO_4_: H_2_O_2_) and distilled water and then dried in an oven at 100 °C. A microplotter needle with an inner diameter of about 200–300 µm was used to rapidly uniformly apply functional ink to the substrate surface. Functional ink was drawn into the needle by capillary action in the amount necessary to ensure a continuous supply of ink throughout the entire printing process. Before the printing process, the needle was brought closer to the substrate surface at a distance of about 10 µm. After that, a powerful pulse of 15–20 V was applied to the piezoelectric element attached to the needle. This causes the excitation of ultrasonic vibrations, which creates a convex meniscus that touches the surface of the substrate. This meniscus maintains stable contact with the substrate throughout the entire printing process. Based on the results of preliminary experiments, it was found that in order to deposit thin films of 1 × 1.5 cm^2^ on quartz substrates, it is necessary to set the following printing parameters: the voltage applied to the piezoelectric element must be 2 V, while the print speed is set at around 10 cm/s. If necessary, additional layers were applied over the first layer in the manner described above.

In our experiment, the colloid of Al NPs with a concentration of 0.18 g/L and ZnO NPs with a concentration of 2.2 g/L in isopropyl alcohol were used as inks to prepare film plasmon nanostructures with different configurations of layers by the microplotter technique. A higher concentration of aluminum and zinc oxide in suspensions for film formation in comparison with luminescence studies in solutions is necessary to obtain films perceptible to the eye, which are characterized by absorption and luminescence, which can be detected by our devices. The use of highly concentrated colloids also significantly reduces the production time of films by reducing the number of layers.

Six samples were produced using similar printing conditions but various procedure sequences. The sample ZnO as a reference luminescence material was pure ZnO nanoparticle film on the quartz template without any metal nanoparticle ensembles. It was prepared by deposition of two printing layers of pure ZnO and characterized by a descending absorption function with a peak at a wavelength of 361 nm and an optical density of 0.08. The Al samples were produced by successive printing of four layers of Al NPs and exhibited an absorption peak at a position of 250 nm and optical density 0.03.

Then, plasmon nanostructures with different combination layers for luminescence enhancement studies were formed. Firstly, the ZnO/Al samples were produced by deposition of four Al layers on the glass template using pure Al colloid as ink, then two layers of ZnO NPs from pure ZnO colloid were applied. Secondly, the Al/ZnO samples were prepared by printing two layers of ZnO on the quartz and then four layers of Al were deposited above ZnO. Thirdly, a sandwich-like structure (Al/ZnO/Al) was formed by the following steps: we deposited two layers of Al NPs on the quartz surface, then two layers of ZnO were printed above Al and, finally, two upper layers of Al NPs were deposited above the ZnO layers.

The sample named Mix (Al+ZnO) was printed using inks containing a mixture of 2 mL of Al NPs colloid and 1 mL of ZnO colloid, which were prepared directly prior to deposition. Six layers of the just-prepared mixture were applied to the quartz substrate. Since in this colloidal mixture the number of ZnO particles is three times less than in the original colloid of zinc oxide with a concentration of 2.2 g/L, six layers of the mixture were necessary to obtain the same surface concentration of zinc oxide in all samples for luminescence investigations. The ratio of number of nanoparticles of zinc oxide to aluminum in the mixed inks approximately corresponds to a colloidal solution of Al 0.011+ZnO 0.22 g/L, where an increase in luminescence up to 1.75-fold (excitation at 300 nm) was observed.

## 3. Results and Discussion

**Figure 2:**
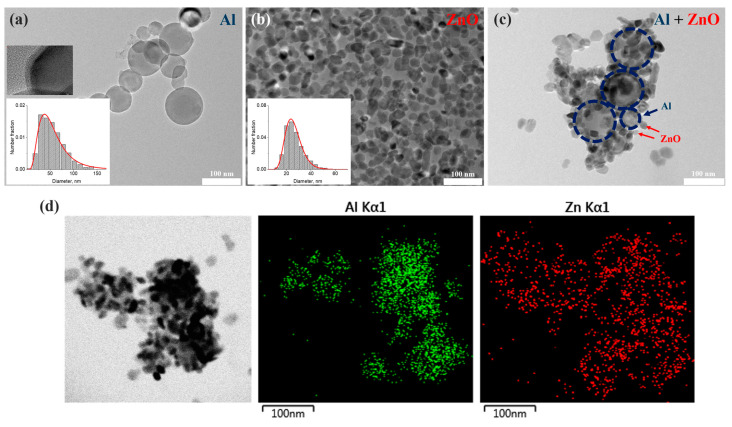
Results of TEM analysis. TEM images of (**a**) metal aluminum, (**b**) zinc oxide nanoparticles and their particle size distributions (inserts), (**c**) an Al-ZnO complex found in the mix colloid of Al 0.0114 g/L + ZnO 0.044 g/L, (**d**) STEM image of an Al-ZnO agglomerate and corresponding EDX elemental map.

The mean hydrodynamic diameter of Al NPs was measured as 140.4 ± 3.1 nm in all colloids despite the concentration of nanoparticles. The obtained value, greatly larger than the average diameter of the nanoparticles resulting from TEM analysis (54.6 ± 25.1 nm), confirms the broad distribution of nanoparticles [51]. The Al nanoparticle ensemble presented in Figure 2a is described by a lognormal distribution with a long tail up to 150 nm, which is typical for gas-phase particles produced by wire explosion [47,49]. Spherical aluminum nanoparticles have a metal/oxide core-shell structure with a crystalline aluminum core and an amorphous aluminum oxide shell (Figure 2a, insert) with average thickness of 3 nm [14], which protects metal nanoparticles from ignition in the natural environment. The as-prepared aluminum suspension with a concentration of ~ 0.26 g/L thanks to citric acid, which provided electric repulsion, turned out to be stable in an alcohol solution. The obtained Al sample retains colloidal stability for at least 30 days. The suspension of Al NPs has negative zeta potential, −29 mV, which confirms the sufficiently good short-term stability to agglomeration.

The colloid of zinc oxide nanoparticles with a concentration of 2.2 g/L has long-term stability for a year [50] and a positive zeta potential of +27 mV. Zinc oxide suspensions diluted by 10–10,000 times are stable for 6 months. ZnO nanoparticles are described by TEM images as an irregular grain with an average diameter of 26.6 ± 7.4 nm (Figure 2b).

The optical density spectra for Al NPs and ZnO NPs colloids with various dilution factors are shown in Figure S1 in the Supplementary Information. The spectra of Al NPs suspensions show a single peak in the UV range, at position of 227 nm for as-prepared and diluted colloids. The shape of the optical density curves of Al and also ZnO nanoparticles do not depend on the concentration of the nanoparticles in solution. The optical density spectrum of ZnO can be described as a decreasing function from 206 to 360 nm with changing slope, a peak at 360 nm and a further sharp drop to almost zero absorption at 390 nm.

### 3.1. Luminescence in Colloids

For the fabrication of plasmon nanostructures, it is important to notice that fluorescence intensity is closely related to distance between fluorophore and metallic nanostructures. To achieve the luminescence enhancement, the emitter nanoparticles are to be located at a specific distance on metal nanoparticles where quenching is still insignificant but electric field and density of state enhancement are still present [6]. This can be achieved by dispersing ZnO nanoparticles with metal nanoparticles at a low concentration and adjusting a spacer between an emitter and the aluminum surface. The optimal distances identified for various metallic nanoparticles and organic fluorophores or quantum dots are considered to be 1–12 nm [6]. So, in our work, to investigate the plasmon-enhanced ZnO luminescence in the presence of metal aluminum nanoparticles, we use low-concentrated solutions of ZnO nanoparticles to obtain negligible effects of self-quenching of luminescence. In addition, a natural aluminum oxide shell plays the role of a dielectric spacer between the zinc oxide and metal aluminum nanoparticles to provide the optimal distance for plasmon enhancement.

Figure S2 in the Supplementary Information depicts the photoluminescent spectra of pure ZnO colloids at various dilution factors. The dilution shifts the position of the PL peak and significantly influences the intensity of the ZnO PL peak. While the colloid of zinc oxide nanoparticles was diluted from 10 to 5000 times, the photoluminescence intensity increased by more than 2000 times. The PL peak at 377 nm in low-concentrated ZnO colloids (0.011, 0.022 and 0.044 g/L) was observed. Moreover, the intensity of photoluminescence grows as a linear function of the number of particles in the solution. Hence, the emission of light is designated by the number of emitters. At higher concentrations of ZnO (from 1.1 to 22 g/L), the position of the PL peak is not stable and shifted from 377 to up to 419 nm, in addition, decreasing the concentration of ZnO results in emission intensity growth. That means the concentration quenching occurs in high-concentrated suspensions, when the ZnO emission is absorbed by ZnO nanoparticles and non-luminescent dimers and agglomerates of ZnO nanoparticles are formed. For the intermediate concentration range of ZnO nanoparticles in solutions from 0.22 to 0.44 g/L, the PL peak position is quite stable (377–380 nm), but the intensity is not a linear function of the concentration.

Since the pure colloids of Al and ZnO NPs have zeta potential of the opposite sign, the surface of metal and semiconductor NPs have an opposite charge. Thus, according to the Coulombs law, we suppose that zinc oxide and metal aluminum nanoparticles are going to attract each other in the mixture of the suspension and form agglomerates.

The colloidal mixtures were obtained by simple mixing of stable initial and diluted solutions of aluminum (with concentrations of 0.057, 0.0285, 0.0114, 0.0057 and 0.00285 g/L) and zinc oxide nanoparticles of various low concentrations (0.022, 0.044, 0.22 and 0.44 g/L) in isopropyl alcohol. It is established that the stability of the colloidal mixture to agglomeration depends on the relative concentration of particles in the solutions and they start to sediment in 5–7 days of storage. The hydrodynamic DLS sizes of nanoparticles in mixture solutions were in the range from 190 to 390 nm depending on the relative concentration. According to the TEM images (Figure 2c), metal and semiconductor nanoparticles create complexes which contain 3-10 spherical aluminum nanoparticles covered by numerous ZnO nanosized grains. EDX maps of several agglomerates represented in Figure 2d confirm the formation of Al-ZnO complexes. The size of obtained complexes varies in a wide range from 130 to 450 nm.

**Figure 3:**
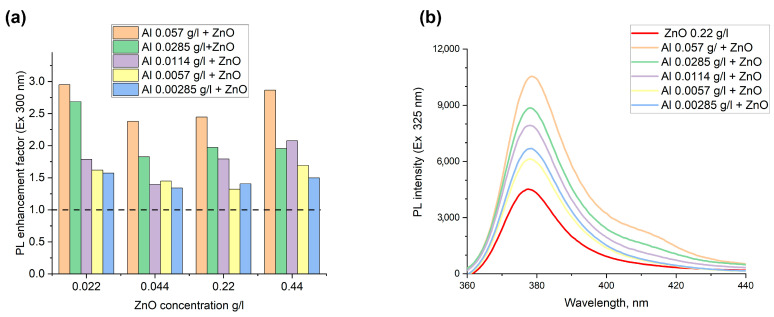
Photoluminescent properties of the colloidal mixture: (**a**) Dependence of the enhancement factor for mixtures with different concentrations of zinc oxide and aluminum oxide nanoparticles; (**b**) Luminescence spectra of pure zinc oxide colloid with a concentration of 0.22 g/L and its mixtures with aluminum colloids of various concentrations (from 0.00285 to 0.057 g/L) at an excitation wavelength of 325 nm.

The increase of the luminescence intensity of zinc oxide nanoparticles in mixtures of metallic aluminum and semiconductor ZnO nanoparticles upon excitation at wavelengths of 300 and 325 nm has been achieved. Figure 3a,b) depict the luminescent enhancement factor for the colloidal mixtures of ZnO and Al NPs and the PL emission spectrum for ZnO NPs of 0.22 g/L with different concentrations of Al NPs. To calculate the enhancement factor, the corrected intensity of the PL peak at 377 nm for a solution mixture of ZnO and Al NPs was divided by the corrected PL intensity of a ZnO suspension with the same fluorophore concentration. Since the colloids have an optical density higher than 0.05, the attenuation of fluorescence due to absorption of the incident light and the emitted light takes place. This fact is called the inner filter effect and can increase or decrease the real photoluminescent intensity. The fluorescence inner filter correction of the colloidal mixture and pure ZnO suspensions according to the formula presented in the paper [52] and Equation (S1) of the Supplementary Information were used.

When the obtained ratio of PL intensity of the mixture to ZnO is more than 1, that means FL enhancement is detected. If the intensity ratio is lower than 1, the quenching effect would be observed. According to the Figure 3, a plasmon enhancement factor of PL in the range from 1.3- to 2.9-fold was found for all colloidal mixtures, despite the concentration of ZnO and Al NPs. Due to the formation of Al-ZnO complexes, observed by TEM, where the metal nanoparticles are located within several nanometers of the fluorophore particles, the excitation electromagnetic wave is focused by metal nanoparticles that consequentially results in luminescence enhancement. The maximum increase in luminescence of up to 3.0-fold at an excitation wavelength of 325 nm and 2.9-fold at the excitation of 300 nm in the colloidal mixtures with the highest concentration of 0.057 g/L for Al NPs and ZnO with a mass fraction of 0.022 g/L was observed. The general trend of increase in the enhancement factor with the growth of concentration of metal nanoparticles was observed for all concentrations of ZnO. An apparent small value of enhancement in solutions with respect to known values for planar plasmon structures based on noble metals is quite significant and important. For instance, up to a 100-fold increase in quantum dots’ emissions on gold nanotriangle arrays and over a 320-fold enhancement for dyes on gold nanoparticles’ arrays were pointed out in the review of [53,54]. Having used silver island films, a 14-fold fluorescence emission increase was achieved, moreover, core-shell silver nanoparticles as substrates showed a 20-fold increase in the fluorescence of dyes and biomolecules [54].

Recent investigations of the interaction of electromagnetic radiation in solutions of mixtures have been already discussed only for organic dyes and noble metal nanoparticles by several research groups. They showed the enhancement in luminescence in the presence of metal nanoparticles in solution not more than eight times. Park et al. showed [55] only 10% enhancement of luminescence in a solution of polydiacetylene polymer of liposomes and gold nanoparticles, as well as the quenching of luminescence depending on the concentration of nanoparticles. Having added 50 nm silver nanoparticles to aqueous solutions of phthalocyanines metallocomplexes, the plasmon resonant enhancement in the fluorescence spectra of the mixture from 1.5 to 7 times was detected by Staruknin et al. [56]. Chu et al. observed an increase in luminescence in the visible region up to 5 times on 20 nm and up to 1.8 times on 100 nm gold particles in colloids of Cy3 dye-doped polystyrene nanoparticles [57]. Notably, the strongest luminescence intensity was reached when the number of fluorophore particles was 10^3^ times more than 100 nm gold nanoparticles and the concentration of metal NPs was 6.4 × 10^7^ particles per mL. With the higher concentration of metal nanoparticles, the fluorescence decreased and quenched, whereas, in our experiments, the maximum enhancement was observed in colloids with a concentration of metal aluminum NPs at 2.5 × 10^11^ particles per ml and 2–30 times higher values for ZnO NPs. At the same time, in the whole range of concentrations of Al NPs (from 1.2 × 10^10^ to 2.5 × 10^11^ particles per ml) and ZnO (from 4 × 10^11^ to 8 × 10^12^ particles per mL), we achieved the enhancement of PL.

### 3.2. Luminescence in Film Nanostructures

Four samples with different layer combinations were fabricated by a microplotter printer using Al and ZnO colloids as inks: Al on the surface of ZnO (Al/ZnO), ZnO on the surface of Al (ZnO/Al), sandwich-like structure (Al/ZnO/Al) and plasmon structure prepared from a colloidal mixture. 

**Figure 4:**
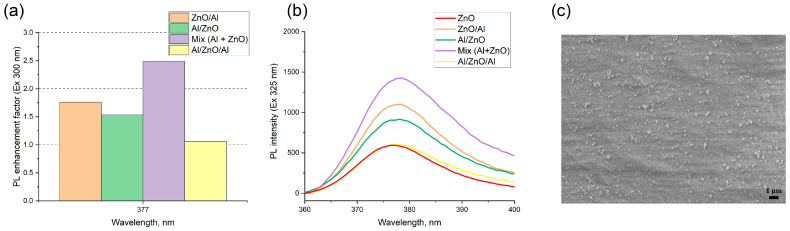
Photoluminescent properties of film nanostructures based on Al and ZnO NPs: (**a**) Dependence of PL enhancement factor at an excitation wavelength of 300 nm for samples with different combinations of layers; (**b**) Luminescence spectra at an excitation wavelength of 325 nm obtained for bare zinc oxide film with a concentration of 2.2 g/L, and fabricated plasmon film nanostructures; (**c**) SEM image of the film fabricated from the colloidal mixture of aluminum and ZnO nanoparticles.

In the luminescence studies, to make use of plasmon enhancement effect, one has to engineer the optimal configuration of an emitter–metal nanostructure to obtain a positive balance of competing enhancement and quenching phenomena. We thoroughly investigated the PL intensities of the pure ZnO, ZnO/Al, Al/ZnO, sandwich-like nanostructures and a film prepared from a colloidal mixture of ZnO-fluorophore and aluminum nanoparticles to understand the amplified electromagnetic effect associated with the interparticle surface–plasmon resonance coupling. The results are presented in Figure 4. The intensity of the ZnO emission sensitively depends on the relative configuration of ZnO and Al nanoparticle layers.

All plasmon structures showed the enhancement of luminescence of ZnO NPs, regardless of the location of aluminum NPs and the configuration of layers, compared with a bare ZnO film. The minimum amplification has a sandwich-like structure, for which the enhancement factor was 1.1. Plasmon structures with successive deposition of aluminum above the ZnO and vice versa have almost the same amplification, approximately 1.8 times for the sample ZnO/Al. The maximum enhancement factor of 2.4-fold was found for a film prepared from a colloidal mixture of the zinc oxide and aluminum nanoparticles. The typical morphology of the obtained plasmon structures is presented on SEM images in Figure 4c. The nanostructure formed from the colloidal mixture of Al and ZnO nanoparticles contains Al-ZnO complexes with sizes from 130 to 330 nm, which occupy only 1% of the surface. That is, due to the usage of the colloidal mixture inks that contain aluminum–zinc complexes, we succeeded in achieving a 28% higher plasmon amplification of the PL intensity than when using the standard procedures for plasmon fabrication such as Al on ZnO, ZnO on Al or sandwich-like structure.

The presented enhancement factor is close to values obtained by other groups on plasmon nanostructures based on Al nanoparticles. A. Polyakov [58] showed a 1.8-fold increase in the photoluminescence and microcathodoluminescence signal from the GaN active region in double heterostructures AlGaN/GaN/AlGaN while adding 100 nm in diameter Al nanoparticles [58]. In 2022, Qiong Ye showed a 2.4-fold enhancement of the band-edge emission of ZnO near cylindric Al NP arrays [29]. The plasmon structure was formed on anodic aluminum oxide templates by a relatively convenient magnetron sputtering method with diameters of metal NPs of 60–100 nm and the space of 125 nm; ZnO film was fabricated by atomic layer deposition on the surface of 15 nm thick aluminum oxide above the Al array. Depending on the Al NPs’ size, enhancement factors of ZnO luminescence at a wavelength of 387 nm from 1.6 to 2.4 were detected [29].

Thus, in our experiments, we observed the comparable values of enhancement factors from 1.1 to 2.4 on random plasmon structures based on Al and ZnO nanoparticles with the size of NPs being 55 and 27 nm, correspondingly.

### 3.3. Numerical Modeling

The enhancement of PL in metal-semiconductor plasmon structures is possible when the enhancing processes provided by the local incident field concentration and radiative decay promotion overcome quenching effects caused by non-radiative metal-induced decay [59,60]. To achieve the maximum photoluminescence intensity enhancement factor, a lot of parameters should be analyzed in numerical modeling such as emission and excitation wavelengths, the spacing between a metal nanoparticle and a fluorophore, the metal’s shape and size, orientation with respect to polarization of incident radiation and the dipole moment of a fluorophore. Using the theory described in papers [60,61] and partly defined in the Equations (S2)–(S4) in the Supplementary Information, we discussed the enhancement of fluorophore luminescence near spherical aluminum nanoparticles of different sizes (2b) with a metal core (2a) in a diameter covered by a dielectric oxide shell with a thickness of 3 nm and a refractive index of 1.81 [62]. The calculation formulas for the case of a nanoparticle with a dielectric shell were carried out in the work [63,64], which used a specified modification of the Mi coefficients. We investigated the distribution of the enhancement factor of PL depending on the spacing (Δr) between the surface of nanoparticles to the fluorophore and the excitation wavelength for an ideal and weak emitter (fluorophore) with quantum yields (Q_0_) of 1 and 0.1.

**Figure 5:**
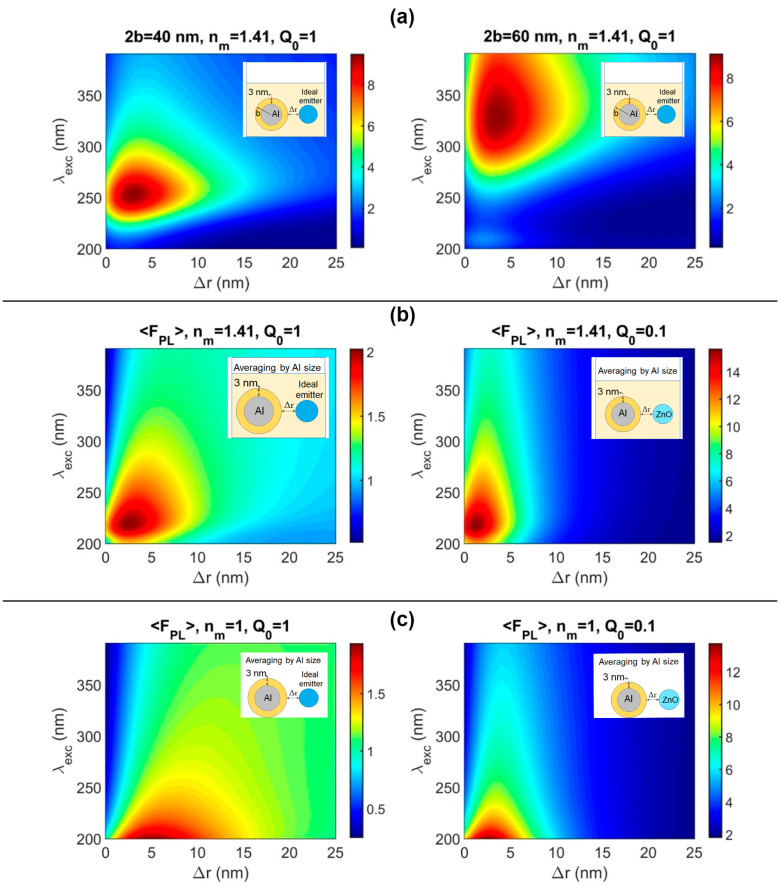
Calculated photoluminescence intensity enhancement factor versus excitation wavelength and metal–emitter spacing for emission wavelength 390 nm for Al nanospheres (**a**) 40 and 60 nm in diameter in isopropanol, intrinsic quantum yield Q_0_ = 1; (**b**) enhancement factor of PL in isopropanol (n = 1.41) averaged over the size of metal nanoparticles and the direction of the electric component of the exciting radiation to the dipole moment of the fluorophore; (**c**) enhancement factor of PL in air atmosphere (n = 1) averaged over the size of metal nanoparticles and the direction of the electric component of the exciting radiation to the dipole moment of the fluorophore with different quantum yields (Q_0_ = 1 and Q_0_ = 0.1).

Figure 5a presents the plasmonic photoluminescence enhancement in the UV environment with aluminum monodispersed spheres with diameters of 40 and 60 nm, which include the aluminum oxide shell at 3 nm in thickness. The ambient medium refractive index was n_m_ = 1 and 1.41, which corresponds to air and isopropyl alcohol, correspondingly. In all calculations (except for cases with field averaging), the transition dipole moment in the fluorophore was orthogonally oriented to the surface of the nanoparticle and parallel to the electric field of the excitation radiation. That means that the case of the maximum possible PL enhancement is derived. One can see the redshift in excitation wavelength to obtain the maximum PL enhancement factor, which ranged from two- to eightfold, and the optimal spacing between the emitter and metal nanoparticle was 2–5 nm.

Since obtained suspensions of Al nanoparticles are polydisperse and have a variation in the size of nanoparticles from 25 to 125 nm according to TEM data, and the vector of the electric component of the exciting radiation is not always parallel to the dipole moment of the emitter, averaging was applied according to the equations described in [58]. Figure 5b shows the enhancement factor of PL (<FPL>) considering the size distribution of Al NPs in suspension (Figure 2a insert) and averaging by the direction of exciting radiation in different media for ideal (Q_0_ = 1) and weak (Q_0_ = 0.1) emitters.

Figure 5b shows that seven-times-higher values of enhancement for an emitter with a weak quantum yield such as ZnO [65] can be achieved. The maximum enhancement factor for the emitter with a weak quantum yield in isopropanol in the presence of Al NPs is up to 14-fold for excitation wavelengths from 200 to 280 nm and the spacing from 0 to 4 nm is realized. For zero spacing between ZnO and Al nanoparticles, excitation wavelengths are 300 and 325 nm as in the above experiment the maximum possible modeling enhancement factors which can be obtained are 4-6-fold for isopropanol and 3–5 times for air. The enhancement factors of 2.4 and 3.0 were achieved in the experiments with Al and ZnO nanoparticle films and colloidal mixtures, correspondingly. The acceptable discrepancy between experimental and modeling data is caused by non-compliance with the condition of one emitter for one metal particle in a real experiment, where we observe the formation of complexes where several Al nanoparticles are covered by numerous ZnO nanoparticles. Thus, we can thoughtfully say that the results of the experimental and modeling investigation are well-comparable.

## 4. Conclusions

We investigated the ultraviolet luminescent properties of a colloidal mixture and film nanostructures based on aluminum and zinc oxide nanoparticles. We prepared colloids of different concentrations of Al and ZnO nanoparticles with average sizes of 54.6 ± 25.1 and 26.6 ± 7.4 nm, correspondingly, in isopropyl alcohol. It was found that the addition of colloids of aluminum NPs with mass fractions from 0.057 to 0.00285 g/L results in plasmon enhancement of ultraviolet luminescence of ZnO nanoparticles at 377 nm in solutions in the range from 130 to 300%. We demonstrated the formation of Al-ZnO agglomerates 130–450 nm in size, which provided the enhancement of UV luminescence in solutions up to 3.0- and 2.9-fold at excitation wavelengths of 325 nm and 300 nm, correspondingly.

Plasmon nanostructures with different constructions of layers, such as Al on the surface of ZnO, ZnO on Al, sandwich structure and samples prepared from a colloidal mixture of ZnO and Al nanoparticles, were fabricated by microplotter printing. We showed that the preparation procedure of film plasmon nanostructures plays a crucial role in the enhancement factor of luminescence. Using a colloidal mixture of metal and semiconductor fluorophore nanoparticles as inks instead of sequential application of pure Al and ZnO colloids allows us to achieve the greatest enhancement factor of 2.4-fold in plasmon nanostructures.

Numerical modeling presented that the enhancement factor for the emitter with a weak quantum yield of 0.1 such as ZnO in the presence of polydisperse Al NPs is calculated to be in the range of 4–6-fold for isopropanol and 3–5 times for air for excitation wavelengths from 300 to 325 nm, which is in good comparability with the obtained experimental results.

The obtained results are a promising boost in the development of low-cost affordable nanostructures based on aluminum nanoparticles fabricated by easy colloidal and printing methods for biomedical and optoelectronic applications in the UV range.

## Data Availability

Not applicable.

## Supplementary Materials

The following supporting information can be downloaded at: https://www.mdpi.com/article/10.3390/nano12224051/s1, Figure S1: UV/Vis Spectra of suspensions with different concentration for (a) ZnO NPs and (b) Al NPs; Figure S2: The fluorescence emission spectra with excitation wavelength 300 (left) and 325 nm (right) for ZnO NPs colloids with different mass fraction of ZnO nanoparticles; Equation (S1): Inner filter fluorescence intensity correction; Equations (S2)–(S4): Theory for numerical modeling.
