# Supplementary material for: Plasmon-Enhanced Ultraviolet Luminescence in Colloid Solutions and Nanostructures Based on Aluminum and ZnO Nanoparticles
Source: Nanomaterials (Basel). 2022 Nov 17;12(22):4051. doi: 10.3390/nano12224051 (PMC9696599; doi:10.3390/nano12224051)
Supplement: Supplementary file 1 [file nanomaterials-12-04051-s001.zip › nanomaterials-2017828-supplementary.pdf]

nanomaterials

MDPI

Supplementary Materials

Plasmon-Enhanced Ultraviolet Luminescence in Colloid Solutions and Nanostructures Based on Aluminum and ZnO Nanoparticles

Anna A. Lizunova [1,*], Dana Malo [1,*], Dmitry V. Guzatov [2], Ivan S. Vlasov [1], Ekaterina I. Kameneva [1], Ivan A. Shuklov [1], Maxim N. Urazov [1], Andrei A. Ramanenka [3] and Victor V. Ivanov [1]

1 Moscow Institute of Physics and Technology, National Research University, 141701 Dolgoprudny, Russia
2 Physico-Technical Department, Yanka Kupala State University of Grodno, Ozheshko Str. 22, 230023 Grodno, Belarus
3 B. I. Stepanov Institute of Physics, National Academy of Sciences of Belarus, Nezavisimosti Ave. 68-2, 220072 Minsk, Belarus
* Correspondence: lizunova.aa@mipt.ru (A.A.L.); malo.d@mipt.ru (D.M.)

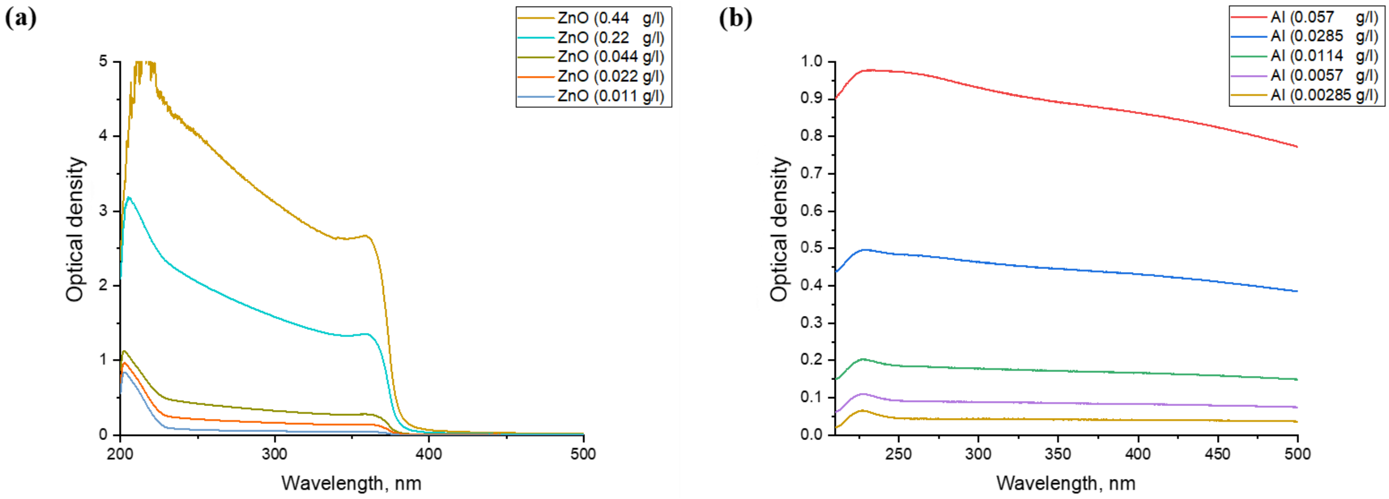

Figure S1. UV/Vis Spectra of suspensions with different concentration for (**a**) ZnO NPs and (**b**) Al NPs.

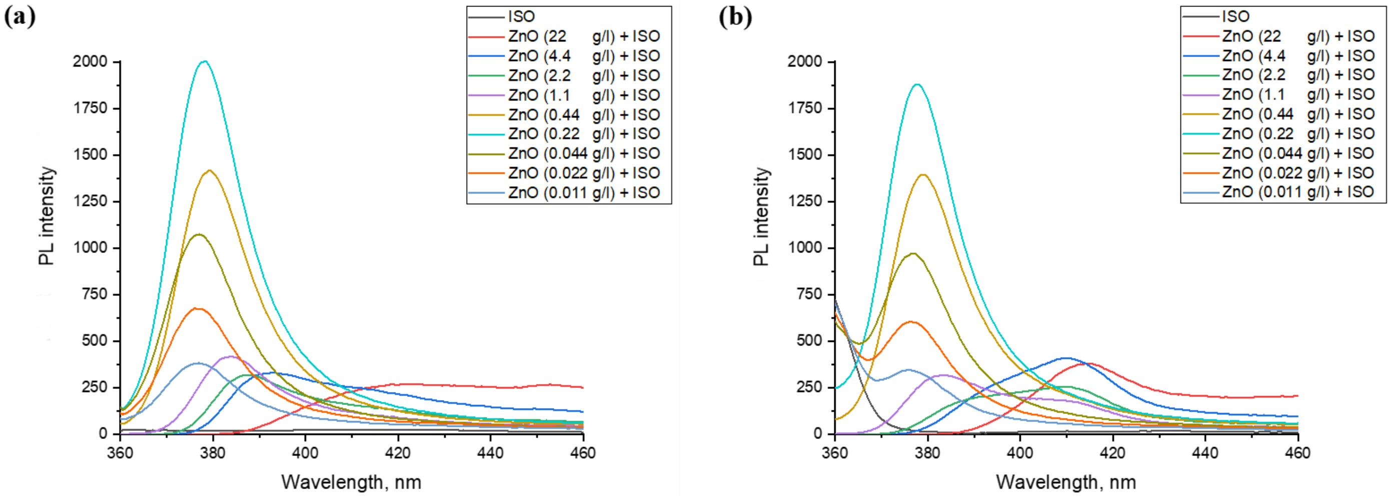

Figure S2. The fluorescence emission spectra with excitation wavelength (**a**) 300 and (**b**) 325 nm for ZnO NPs colloids with different mass fraction of ZnO nanoparticles.

Inner Filter Correction

If the sample has a significant optical density at both the excitation and emission wavelengths, OD_{ex} and OD_{em}, respectively, these optical densities attenuate the excitation and emission light, that is called the primary and secondary inner filter effects, respectively.

The corrected fluorescence intensity is given approximately by [1]

$$F_{corr} = F_{obs} antilog\ (\frac{OD_{ex} + OD_{em}}{2}) \quad \text{(S1)}$$

where

F_{corr}—corrected intensity of photoluminescence
F_{obs}—intensity of photoluminescence observed in the experiment
OD_{ex}—optical density of the sample at the excitation wavelengths
OD_{em}—optical density at the emission wavelengths

Theory for Numerical Modeling

Considering that the luminescence intensity is proportional to the intensity of the absorbed radiation and the quantum yield, and assuming that the absorption coefficient of radiation by a molecule (nanocrystal) in plasmon structures does not change, we can write an expression for the luminescence intensity change factor of an elementary emitter F_{PL} at a point with a radius vector r in the following form [2,3]:

$$F_{PL}(\omega, \omega', r) = G(\omega, r)Q(\omega', r), \quad \text{(S2)}$$

where ω (ω') –frequency of exciting and emitting radiation;

$$G = \frac{|E(\omega, r)|^2}{|E_0(\omega)|^2} \quad \text{(S3)}$$

G—the enhancement factor of intensity of the electric field of exciting radiation at a given point r, in which E/E_0 – is electric field amplitudes in the presence of a metal nanoparticle and without it.

Q is a quantum yield of luminescence near the nanoparticle [3], which can be described as

$$Q = \frac{\gamma_{rad}(\omega', r)}{\gamma_{rad}(\omega', r) + \gamma_{nonrad}(\omega', r) + \gamma_{\text{int}}(\omega')} \quad \text{(S4)}$$

where γ_{rad} (γ_{nonrad}) – radiative (non-radiative) transition from the excited to the ground state of a quantum system; γ_{int} is nonradiative decay, describing the losses in the emitter. It doesn't depend on the presence of nanoparticle and can be expressed by intrinsic quantum yield Q_0 of the molecule: $\gamma_{int}=\gamma_0(1/Q_0–1)$, where γ_0 – radiative transition in the emitter without the metal nanoparticle.

Further, only the case of the orientation of the dipole moment of the molecule normal to the surface of the nanoparticle will be considered, since in this case the F_{PL} factor may increase [2-4]. To coordinate the factors G and Q in (2), we suppose that the dipole moment of the molecule is oriented in the direction of the electric field strength of the exciting radiation.

The enhancement factor G is calculated by solving the case of the Mi scattering of a linearly polarized plane electromagnetic wave on a spherical particle with a shell [5]. The expressions for the factor G can also be obtained from the expressions in [2] for a solid metal particle by replacing the coefficients Mi for the scattered field with the corresponding coefficients Mi for a particle with a shell [4,5]. These expressions are not explicitly given here because of their cumbersome aspect.

To find the quantum yield Q, both the radiation and radiation components of the rate of spontaneous decay of a molecule near a spherical metal particle should be calculated. In this case, the non-radiative component of the decay rate can be calculated as the differ-

ence between the total (γ_{tot}) and radiation rates, i.e. by the formula: $\gamma_{nonrad}=\gamma_{tot}-\gamma_{rad}$. The explicit expressions for the relations γ_{tot}/γ_0 and γ_{rad}/γ_0 in the case of a molecule near a spherical particle with a shell can be found in [4].

In our calculations, a nanoparticle with an aluminum core (Al) and a dielectric shell (Al_2O_3) was discussed. Experimental data on the dependence of the dielectric permittivity of aluminum on the wavelength were taken from the reference book [6]. The refractive index of the shell was set to 1.81, and the thickness of the oxide shell was 3 nm. The full diameter of the particle with the shell was further indicated as 2b, and the distance from the emitter to the surface of the layered nanoparticle was indicated as Δr .

References

1. Lakowicz, J.R. *Principles of Fluorescence Spectroscopy*, 3rd ed.; University of Maryland School of Medicine: Baltimore, MD, USA, 2006.
2. Guzatov, D.V. et al., Plasmonic enhancement of molecular fluorescence near silver nanoparticles: Theory, modeling, and experiment. *J. Phys. Chem. C* **2012**, *116*, 10723–10733.
3. Guzatov, D.; Gaponenko, S.V.; Demir, H.V. Colloidal Photoluminescent Refractive Index Nanosensor Using Plasmonic Effects. *Z. Phys. Chem.* **2018**, *232*, 1431–1441. https://doi.org/10.1515/zpch-2018-1127.
4. Gaponenko, S.V.; Adam, P.-M.; Guzatov, D.; Muravitskaya, A.O. Possible nanoantenna control of chlorophyll dynamics for bioinspired photovoltaics. *Sci. Rep.* **2019**, *9*, 7138. https://doi.org/10.1038/s41598-019-43545-4.
5. Bohren C.F., Huffman D.R. *Absorption and Scattering of Light by Small Particles*; John Wiley-Interscience: New York, NY, USA, 1983; 530p.
6. Palik, E.D. (Ed.) *Handbook of Optical Constants of Solids*; Academic Press: New York, NY, USA, 1998; 805p.
